# Bad Deals and Tragic Pacts: The impact of the EU’s management of migration through the externalisation of its borders on ‘Women on the Move’

**DOI:** 10.12688/openreseurope.18388.1

**Published:** 2024-08-28

**Authors:** Mary Grace Vella

**Affiliations:** 1 Faculty for Social Wellbeing, Department of Criminology, University of Malta, Msida, Malta

**Keywords:** Female migration, EU-Turkey Deal, New Pact on Migration and Asylum, human rights

## Abstract

In recent years the EU has become increasingly restrictive in its regulation of irregular migration flows despite its outward sustained discourse of safeguarding human lives and the protection of fundamental rights and freedoms. As part of this restrictive strategy, the EU has invested heavily in the securitisation and the management of migration through the externalisation of its borders. The EU-Turkey Deal struck in 2016 paved the way for further agreements with third countries with poor gender equality and human rights records, such as Libya and Tunisia. This externalisation of migration constitutes an important feature of the New Pact on Migration and Asylum adopted in 2023 with the aim of establishing a common asylum process at EU level. Asylum seekers, particularly girls and women are placed at added risk from these restrictive policies and policies of externalisation, as apart from dangers faced by all asylum seekers, they are at increased risk of human trafficking, sexual abuse and exploitation, and others forms of gender-based violence. Despite offering a short-sighted measure to a complex problem and the adverse humanitarian repercussions and gross human rights violations, particularly on female refugees and asylum seekers, such deals and pacts constitute an integral aspect of the EU’s strategic agenda of managing migration through the externalisation of its borders. The article proposes a number of alternative solution-oriented measures which safeguard fundamental human rights and freedoms, in particular the right to asylum for refugee women and girls.

## A Refugee law in crisis

Globally “ethnic tensions, political strife, famine, climate change and terrorism continue to uproot lives…The number of people seeking refuge outside of their home countries or displaced within is higher than ever - and it's women and girls who are suffering the most” ( Women for Women International, 2023, para. 2).

Yet, at such time of need, “international refugee law is in crisis” with various nation states relinquishing their obligations to provide protection, more often instead “committed to a pattern of defensive strategies designed to avoid international legal responsibility” ( Hathaway & Neve, 1997, p.115–116). The EU-Turkey deal is a case in point.

Indeed, the main objective of the deal was to deter the crossing of third country nationals across EU borders and discourage asylum seeking migration to Europe. Despite its human rights violations and negative repercussions on the well-being of migrants and asylum seekers, particularly on women and vulnerable groups, the EU-Turkey deal remains a distinctive feature of the EU’s externalisation of its migration policy as it continues to make agreements and provide funding and other resources to non-EU states, including those with poor human rights records. This coordinated strategy has indeed “unleashed some of the most severe human rights impacts” contributing to a drastic increase in death rates whilst presenting third nations “with more leverage to negotiate their own interests” ( Casajuana & Pintus, 2023, p.7).

Half of internally displaced, stateless people and refugees are girls and women ( Women for Women International, 2023). Female asylum seekers and migrants are each personally, racially, and culturally diverse. Yet, the feminisation of migration is closely aligned with the feminisation of poverty ( Russell, 2014), gender discrimination, and victimisation from life-threatening mental and physical trauma ( Setia *et al.*, 2011), and sexual abuse and violence ( Albrecht *et al.*, 2021). Thus, in addition to the challenges arising from the migratory experience, they confront additional gender-specific challenges leading to an “added layer of oppression” ( Women for Women International, 2023, para. 13). Nonetheless, “their voices are often left out of policies that are designed to protect them” ( Women for Women International, 2023, para. 13) resulting in a situation where their needs and realities are often overlooked, and injustices perpetrated. Women and girls are primary victims of the EU-Turkey deal as their human rights, including the fundamental rights to asylum are arbitrary violated. An alternative solution-oriented approach to the EU-Turkey deal thus demands a holistic approach to the well-being of migrant women and girls.

## Female integration and wellbeing

“Protection, if carefully designed and delivered, is the critical complement to root causes intervention” ( Hathaway & Neve, 1997, p.211). Thus, apart from preventative proactive action to address the pressures of involuntary migration, integration and support initiatives need to be consolidated for those who notwithstanding the externalisation of the EU’s migration policy manage to safely arrive within the jurisdiction of the EU. While for all genders, integration is activated through the capacity to exercise one’s rights enshrined in international protection, and thus dependent on “legal protection in the process of integration of refugee status determination process” ( Jaji, 2009), compared to their male counterparts, female asylum seekers tend to face greater and possibly more complex integration challenges. This is because “it is not only language, culture, access to work and western school systems that present obstacles. Women are traditionally also confronted with additional household and childcare duties. Their challenges are thus twofold” ( Albrecht *et al.*, 2021, p.39).

Moreover, given the patriarchal nature of societies in both countries of origin and countries of destination, female migrants are subject to various discriminatory practices, including of a legal and institutional nature on the basis of sex and gender.

Apart from being embedded in non-discriminatory practices, the effectiveness of integration measures largely depend on integrated service provision across all areas of well-being, including health, housing, education, employment, and socio-cultural support and participation. Such constituents of wellbeing are safeguarded under Article 14 of the 1948 Universal Declaration of Human Rights, which decrees that: “Everyone has the right to a standard of living adequate for the health and well-being of himself and of his family, including food, clothing, housing and medical care and necessary social services, and the right to security in the event of unemployment, sickness, disability, widowhood, old age or other lack of livelihood in circumstances beyond his control” ( UN General Assembly, 1948). Yet, female migrants and asylum seekers face various challenges in accessing quality services across all areas, primarily due to issues of accessibility, availability, adequacy and affordability of services. Apart from legal eligibility impediments, barriers might include gender roles and care responsibilities, language and cultural hurdles, inflexible service provision, and lack or limited awareness of available services and their entitlement ( De Maio *et al.*, 2017). Another issue which impacts the uptake of entitled services is reliance on informal support networks, rather than access of formal service provision ( De Maio *et al.*, 2017). Challenges and barriers tend to be more acute in the uptake of specialist, rather than mainstream services ( De Maio *et al.*, 2017). Discriminatory treatment is also a significant challenge for accessing services by female migrants as it is often impacted by institutional forms of racism and patriarchy.

Lacunae in service provision and discriminatory institutionary practices are often justified on the basis of crisis intervention, in the conviction that asylum seeking forms of migration are of a temporary, rather than of a permanent or long-term nature. However, reality shows that a significant proportion of asylum seekers including those who have been granted a temporary protection status will not be able to return to their homes in the immediate future ( Lokshin & Olters, 2022). This validates further the need for the provision of effective high-quality integration policies and services especially for female migrants and asylum seekers who are often “first responders and transfer their experiences directly to their children” ( Albrecht *et al.*, 2021, p.39).

Integration entails experiencing a sense of belonging. For this purpose, “safety and security of housing” constitutes a determining factor in asylum seekers’ sentiment of “feeling ‘at home’” ( Pertek *et al.*, 2018, p.13). Yet, homelessness and housing insecurity and exclusion is a significant reality amongst migrants and asylum seekers, which acts as a major barrier to access other important rights and services. 

Home is also intrinsically connected to family relationships and significant others. Family reunion is a significant influencing factor for integration, since it impacts on the opportunities of migrants to rebuild their lives, on social networks, and support structures, which in turn impact all areas of socio-economic and psychological wellbeing. Family separation and barriers to reunion constitute an important contributor to mental health difficulties amongst migrant communities ( De Maio *et al.*, 2017). Yet, most EU countries have adopted a resolute restrictive approach to family reunification, despite this being protected under international human rights law. In view of this, the EU needs to consolidate its humanitarian family reunification policies and programs by improving access to reunion through greater flexibility in eligibility criteria, required documentation, concessions and waiving of application and processing fees, and assistance in settlement programmes.

Being physically and psychologically ‘healthy’ is another important factor for successful integration ( Ager & Strang, 2008). Physical and mental trauma often experienced by asylum seekers and refugees increases risks of health difficulties, morbidity, and lowered life expectancy ( Setia *et al.*, 2011; UNHCR, 2011). Female refugees and asylum seekers are subject to various health issues, in particular issues relating to maternal wellbeing, chronic diseases including diabetes, hypertension, and cancer, and gender-based violence ( Ali *et al.*, 2004; Shaw, 2009), injurious traditional practices ( Fathalla *et al.*, 2006; Lawrence & Kearns, 2005), as well as stress-related difficulties ( Ohene-Bekoe, 2017). These health issues become augmented through external influences such as lack or limited access to health care, and conditions in detention and refugee camps ( Ali *et al.*, 2004). Moreover, access to health care is also impacted by knowledge of existing services ( Setia *et al.*, 2011), and long waiting times ( Setia *et al.*, 2011) with quality-of-care access best appraised through its uptake ( Setia *et al.*, 2011).

Fear and misinformation may also impact access to health ( Fathalla *et al.*, 2006; Lawrence & Kearns, 2005), as are language proficiency and cultural beliefs ( Ohene-Bekoe, 2017). Indeed, asylum seekers and refugees’ health issues are impacted by both proximal factors or daily stressors (such as poverty and social exclusion), and distal factors, characterised by occasional or sporadic events (such as a traumatic event) ( Miller & Rasmussen, 2010). Successful integration thus demands access to “timely, high-quality health care” ( Ohene-Bekoe, 2017, p.48).

Education ( Akua-Sakyiwah, 2016; Gesemann, 2006), in particular language competency ( Deacon & Sullivan, 2009) is another important constituent for integration as it enables understanding about rights and services as well as facilitates employment prospects. Despite that both education and work are guaranteed fundamental rights, female migrants and asylum-seekers tend to face many barriers from officially accessing quality education and employment in the host country ( UNICEF, 2017), with various negative repercussions on their wellbeing, as they often end working in the informal economy under precariat conditions and exploitative practices. Training with particular focus on language competency ( Namak *et al.*, 2022) and “access to safe and lawful work” including in the digital economy ( Nicolle, 2022), supports migrants and asylum seekers to “contribute to the needs of their family, community and the country in which they reside” and “become an economic and social boon to their host countries” ( UNICEF, 2017, p.3). These opportunities are particularly warranted for female migrants, as apart from the fact that “gender discrimination closes doors or leads to lower pay” ( Women for Women International, 2023, para. 20), gendered work areas and environments such as domestic work, care work and sex work are subject to greater risk and exploitative practices, including isolation, lack of health and safety standards, gender pay gaps and vulnerability to physical and sexual abuse. The integration of female asylum seekers and refugees in quality work not only impacts their wellbeing and helps to “close gaps in poverty, gender equality, and inclusive work” but also sustains “economies on a local and global scale” ( Women for Women International, 2023, para. 20) as annually they conceivably contribute to $1.4 trillion to the global GDP ( Women for Women International, 2023, para. 18).

Such measures which impact both the affective and functional dimensions ( Wessendorf & Phillimore, 2018) should help to instil a sense of connectedness and belonging which is pivotal for integration ( Ager & Strang, 2008; Cheung & Phillimore 2017). The development and implementation of any integration and support initiatives for female asylum seekers and migrants need to be embedded within a trauma sensitive and informed approach. As earlier recalled, violence and abuse against female asylum seekers is “widespread and systematic” ( Amnesty International, 2021) and “spatially and temporally complex” (Pertek *et al.*, p. 25) as it traverses across all stages of the migratory experience. Indeed, as “trauma could occur pre-conflict, during conflict, in flight and in refuge” ( Pertek *et al.*, 2018, p. 25), it needs to be “conceptualised as an ongoing and multi-faceted experience” ( Pertek *et al.*, 2018, p. 6). Such traumatic experience characterised by dehumanizing and discriminatory treatment includes sexual and gender-based violence, kidnapping and abduction for ransom, human trafficking, child marriage and physical and sexual violence and slavery ( Amnesty International, 2021; Casajuana & Pintus, 2023, p.29). The prevalence of sexual and gender-based violence is rampant, as one fifth of all displaced women and refugees are subject to such forms of violence ( Vu *et al.*, 2014), with another study reporting a prevalence of up to 69.3% of female migrants (as compared to up to 28.6% of males) ( Keygnaert & Guieu, 2015). Despite the varied and multiple physical, psychological, emotional and social aftermaths in both the short and long term ( Tavara, 2006), “affecting overall wellbeing” ( Pertek *et al.*, 2018, p. 11), yet it remains the case that “mainstream integration scholarship” ( Pertek *et al.*, 2018, p.11) and services do not give adequate attention to sexual and gender-based violence, with the result that many migrant survivors slip through the cracks of the support system. Effective integration work thus demands building the capacity of frontline workers to recognize trauma and abuse and be empowered to support victims ( Mwenyango, 2023). 

## Heterogeneity and Intersectionality

Though as a population, female migrant and asylum seekers uphold specific needs due to their gendered realities, their heterogeneity leads to “uniqueness in individual circumstances, experiences and opportunities” ( Jaji, 2009, p. 223). Recognition is also made of the individualised nature and complexity of migration trajectories, as well as its dynamic, non-linear and non-unidirectional movement from countries of origin to countries of destination ( Snel *et al.*, 2021).

Intersectionality acknowledges the interdependence of the “various factors that influence the process of integration” ( Jaji, 2009, p.219), including the legal, political, economic, social, and cultural realms. Due recognition of this interdependence and interconnectedness between these multiple dimensions is pivotal for the adoption of “a holistic approach” to integration ( Jaji, 2009, p.219). Moreover, an intersectional approach appreciates that “the objective aspects of integration are mediated by subjective factors” that shape the different realities and experiences of women ( Jaji, 2009, p.230). It also acknowledges that these intersectionalities can exacerbate existing difficulties and challenges. For example, for female asylum seekers, sexual and gender-based violence intersects with the vulnerability arising from their refugee status as this intensifies victimisation and leaves limited avenues for redress. As a result, such violence becomes particularly salient because of “its targeted nature” ( Jaji, 2009, p. viii).

An additional challenge with existing policy development and implementation frameworks is that most service provision is developed within a crisis intervention and ‘temporary’ protection approach with little focus and opportunities for sincere integration efforts. Yet, the prolonged crisis in the country of origin and protracted statuses of displacement, result in many asylum seekers living in limbo - “denied the right to integrate in the asylum state, yet are unlikely to be restored to meaningful membership in their home community” through successful voluntary repatriation ( Hathaway & Neve, 1997, p.130–131). This situation jeopardises asylum seekers’ psychosocial needs and wellbeing, leading to “uncertainty and despair” as they “are unable to return home and yet are ineligible for permanent membership in their asylum state” ( Hathaway & Neve, 1997, p.132).

Another main failure of existing policy frameworks is intrinsically linked to their governance mechanisms, characterised by top-down interest driven approaches. Despite EU discourse about participation, engagement and ownership by those most affected, migrants and asylum seekers’ engagement needs to “move away from symbolism and tokenism” ( Ramazani, 2023, p.28) towards sincere and “meaningful participation, efforts to move toward co-design, ownership, and eventual transfers of decision-making power” ( Ramazani, 2023, p.28). Meaningful and sincere engagement of diverse groups of migrants and asylum seekers in governance mechanisms and proceedings would ensure that their heterogeneity, experiential knowledge, and expertise is acknowledged and reflected in policy making processes and service provision.

## Embodied inquiry

As the EU-Turkey Deal and the externalisation of migration has become an established feature of EU policy despite its negative human rights and humanitarian repercussions, it is imperative that further analysis is sustained on the impact that such praxis has on both the individual and structural level.

In particular, examination is needed on how the ‘new’ EU Pact on Migration and Asylum ( European Commission, 2023) will impact the EU-Turkey deal given the new ‘burden-sharing’ mechanism across EU countries. This analysis needs to specifically consider a gender impact assessment of the repercussions of such agreements. Its encapsulation of the voices and experiences of female migrants and asylum seekers is pivotal to capture and gain insight of its impact on the embodied realities on migrant's lives.

In addition to being developed and implemented in a gender responsive and culturally appropriate manner, strategies need to build on evidence-based practices, assessment of best practices, and ‘strengths-based’ models of service delivery ( De Maio *et al.*, 2017, p.46) whilst addressing existing gaps in relation to services and programs. In addition, it is pivotal that sensitivity to intersectional issues are addressed “to understand the differences in needs, outcomes and experiences of refugees of all genders and ages regardless of their background” ( Pertek *et al.*, 2018, p. 27). This demands fostering collaborative relationships with migrant and refugee communities in order to better understand the needs and realities of female migrants.

While a wider knowledge-base helps to further appraise migratory experiences, the evidence to inform integration policies, services and programs for female asylum seekers and migrants is clear and extant. It points not only to personal circumstances which may lead to vulnerabilities and victimisation, but in particular to structural deficiencies in both countries of origin and host communities. Such structural deficiencies which speak of discrimination, inequalities, and oppressive and exploitative practices, including institutionalised racism and sexism, continue to shape the realities for female asylum seekers and migrants. This thus demands the transformation of discourse and evidence into concrete action. 

## The new pact on migration and asylum: Another tragic pact for women on the move

Despite EU discourse of human rights, rule of law and solidarity, many lives are still tragically lost in dangerous migratory processes across the Mediterranean Sea whilst seeking safety, protection, and a better quality of life into Europe. Many other migrants and asylum seekers face detention, inhumane and degrading treatment, exploitation by smugglers and human traffickers, and repatriation and pushbacks by governments. For those who make it into Europe, the challenges are far from over as they continue to face exclusion, ongoing discrimination, racism, hate crime, and exploitative practices.

Unfortunately, the “international refugee protection system serves fewer and fewer people, less and less well, as time goes on” ( Hathaway & Neve, 1997, p.137). The New Pact on Migration and Asylum (hereafter referred as the Pact) reached on 20 ^th^ December 2023 continues this austerity trend, and whilst “hailed in Brussels as a great European success, nevertheless, leaves unresolved some problematic aspects” ( De Petris, 2023, para. 4).

The original scope of the Pact was that of addressing the fragmented, isolated, and often ad hoc initiatives to respond to migration flows to the EU by individual Member States. The Pact aimed to redress this problem of individuated state action by providing a joint EU response binding all Member States to act in solidarity with those states on the external European border such as Greece, Italy, Malta and Spain by ‘sharing the burden’ of migration. The Pact thus aimed at regulating primarily the EU 'internal dimension' of migration, whilst complementing its 'external dimension' of migration management.

The Pact endorsed five main measures, relating to the Screening Regulation ^1^, which establishes a pre-entry method for the expeditious profiling of migrants and asylum seekers; a revised Eurodac Regulation to prevent different requests under the same name; a revised Asylum Procedures Regulation to enable a more rapid border procedure (up to 12 weeks) along with the customary lengthier asylum procedure; the introduction of the Asylum and Migration Management Regulation which introduces ‘compulsory solidarity’ on a flexible and discretionary basis to support other EU countries affected by large migratory flows ^2^; and the Crisis and Force Majeure Regulation which provides for the application of stricter migration management rules (such as longer detention periods) in cases of massive asylum flows.

The Pact has been severely criticised for entrenching “outdated ideas on how to deal with migration and fail to take into account the reality at the EU’s borders both on the ground and in the sea” whilst undermining “the right to asylum, international law and human rights” ( Lamberts, 2023, para 3).

While making some strides in solidarity among member states for ‘burden’ sharing, however the ‘compulsory’ yet flexible and voluntary nature of this solidarity mechanism “seems almost an oxymoron” ( De Petris, 2023, para. 22). Moreover, enabling countries to pay a monetary contribution instead of migrant relocation, neither helps to “create a real network of solidarity within the Union” ( De Petris, 2023, para. 22), nor addresses the challenges faced by those countries which receive large concentrations of migrants. 

The Pact has also been severely criticised for its deficient protection of the fundamental right of asylum, due to its introduction of the use of an accelerated procedure for examining asylum applications. Moreover, being primarily concerned with border and migration management, the Pact sustains the securitization of migration wherein the humanitarian aspects of migration is “negotiated against pre-entry screenings of individuals, reception and resettlement procedures” on the grounds of security issues ( Standke-Erdmann, 2021, pg.20).

In terms of the concerns raised on the EU-Turkey deal, a main failure of the pact is that by strengthening existing border management actors, it will “further progress” the externalization of migration, leading to “(re)produce forms of discrimination and violence, as it will be increasingly complicated to hold actors accountable for human rights violations or systemic discriminatory behaviour” ( Standke-Erdmann, 2021, p.20). While focussing on the EU internal dimension, the Pact consolidates the externalisation of migration management through agreements and economic support with third countries outside the EU with shady track record of fundamental rights. This will thus see the sustained systematic detention, violence and abuse of migrants with particular damaging impact on female migrants and vulnerable groups.

Indeed, the Pact has been criticised for its reproduction of gendered, racialized, and ableist stereotyping of migrants ( Standke-Erdmann, 2021) “which leave no room for people’s agency and the adaptation to individual situations and needs” ( Standke-Erdmann, 2021, p.19). Moreover, the pact fails to adopt an intersectional approach “at a normative, policy or institutional level” or within its “suggested measures and procedures” ( Standke-Erdmann, 2021, p.19).

Due to these issues, the agreement has been criticised for offering “a pact at all costs that undermines the credibility of the EU” ( De Petris, 2023, para. 20) and departs from its “principles and values of which it claims to be the proud defender” ( De Petris, 2023, para. 25).

The pact will thus further endanger the lives and wellbeing of asylum seekers and refugees particularly of female migrants and other vulnerable groups through sustaining systematic detention in third countries with poor human rights records, undermining procedural standards and safeguards of asylum procedures, and sustaining the outsourcing of migration management to third countries without adequate monitoring and scrutiny. Indeed, “funding autocrats and militias to handle migration management without any democratic scrutiny or fundamental rights is a recipe for abuses and attacks against people's liberties” ( Greens/EFA, n.d., para.32).

The Pact indeed goes two steps back from the agreements made in the ‘Global Compact for Safe, Orderly and Regular Migration’ ( UN, 2018) which also aspired towards a common approach to international migration. The global compact, comprising 23 objectives for the improvement of migration management in nations of origin, transit and destination and at local, national, regional and global levels, sought to a) mitigate against adverse factors that inhibit local sustainable livelihoods; b) reduce risks and vulnerabilities faced by migrants throughout the migration experiences within a human rights and supportive framework; c) recognize the, economic, social, environmental and demographic transformations that may impact and result from migration; and; d) develop conducive environments to enable the enrichment of societies through migration. The compact also adopted a gender responsive and gender mainstreamed approach by emphasizing gender equality and female empowerment whilst “recognizing their independence, agency and leadership in order to move away from addressing migrant women primarily through a lens of victimhood” ( Lochan *et al.*, 2019, p.36). Despite these noble objectives underlining a human rights approach and cooperation and solidarity, being non-legally binding, the compact leaves migrant protection in the hands of state sovereignty.

Notwithstanding various calls by the humanitarian global community for respect for international law and protection of fundamental rights, “solidarity now means border surveillance within the EU, relocation is not prioritised and there are no specific solidarity procedures for search and rescue disembarkation” ( Lamberts, 2023, para 5).

While the initial scope of legal and policy instruments was that of ensuring that refugee protection couldn’t be left to political discretion and humanitarian philanthropy, it is now becoming increasingly acceptable that supranational institutions themselves are enabled to act inhumanely outside the rubric of international law. Indeed, “instead of seeking humane and practical solutions to manage migration in an orderly and safe way, EU Member States have been pursuing dangerous policies that are turning the Mediterranean into a graveyard” ( Lamberts, 2023, para 3).

Given Turkey’s history of sustained violations of human rights, including the poor protection of women’s liberties, Turkey and any other countries where fundamental human rights are not guaranteed should not be considered as legitimate partners in the humanitarian ‘management’ of migration. It is imperative that such deals are relinquished, and true alternatives established that offer safe passages to Europe for asylum seekers, in full protection of fundamental rights and freedoms.

The EU has and continues to fail from providing safe routes for humanitarian migration. At the same time, it also fails from taking proactive action to prevent migration by addressing its root causes. As acknowledged by the UNHCR (1995, p. 43), “refugee movements are not inevitable, but can be averted if action is taken to reduce or remove the threats which force people to leave their own country and seek sanctuary elsewhere.” This demands individual and collective state action to address the issue through the pursuance of political, economic, and humanitarian agendas ( UNHCR, 1995).

“Migration is a manifestation of inequality. However, its structural causes in a capitalist, globalised world are not analysed” ( Dağdelen, 2018, para. 18), acknowledged, let alone addressed. Indeed, current practices as evident from the EU Turkey Deal and the New Pact for Migration continue to consolidate this structural inequality as migration, framed within the false discourse of human rights becomes another tool for the control and oppression of the rich and powerful over the poor and destitute. 

The EU needs to adopt a more proactive approach towards the prevention of involuntary migration, such as those arising from wars and conflicts and violations of human rights and persecution. It also needs to take a more proactive approach to address migratory processes arising from economic and environmental vulnerabilities in countries with high levels of net migration.

This however demands a new economic and political order. It demands a sustained commitment to end the armaments industry and its exports, which fuels wars and conflict, and which profits from the ‘border militarization’ against irregular migration ( de Vries, 2022). It indeed demands recognition that displacement does not take place in a vacuum but is often

the consequence of decisions taken by powerful individuals and institutions, both within and outside of the countries which actually produce refugees. Wars do not start unless an army is ordered into action… Ethnic cleansing only takes place when political leaders and their supporters conclude that it is in their interest to organize such expulsions. ( UNHCR, 1995)

Apart from relinquishing its vested interests in the production and sales of weapons of war, the EU can play a pivotal role as an international mediator for peace - “through its political and financial weight and its comprehensive approach to conflict prevention and resolution, involving CFSP/ESDP and Community instruments.” It is indeed “in an excellent position to provide incentives to the conflict parties and can rely on its wide field presence” ( Council of the European Union, 2009, p.4). Yet, as evident from the ongoing occupation of Palestinian territory and the genocide of people in Gaza, the EU often shuns away from taking such preventative proactive humanitarian action.

Such proactive action also demands a commitment to stop the exploitative transfer of resources from south to north – which wholly benefit the North but continues to oppress and impede the South from autonomous development. Whilst the exploitation of the South through the uptake of human resources results in brain-drain which “paralyses economic development” ( Dağdelen, 2018, para. 12), it benefits countries of destination through more extensive labour power – acting as a reserve army of labour, to keep wages low and work precarious. Indeed, it is estimated that up to 50 per cent of those academically educated emigrate from the South ( Dağdelen, 2018). Yet, despite these benefits of migration to the host countries, many “Member States continue to misuse the issue as a scapegoat for societal challenges” ( Greens/EFA, n.d., para.2). A new economic model demands a reevaluation of current practices of free trade agreements ( Dağdelen, 2018) which sustain ‘Open Trade’, yet ‘Closed Borders’ for immigration ( Peters, 2015). Proactive measures also entail supporting countries to overcome long-term developmental challenges in line with the 2030 Agenda and its Sustainable Development Goals ( Casajuana & Pintus, 2023, p.53).

The EU is in dire need of adopting a migration policy which “treats people seeking safety with dignity…. enables safe passage, upholds human rights law and facilitates successful integration” ( Greens/EFA, n.d., para.2). It demands a shift from current mainstream discourses of migration as an issue of national security towards one of human security, which does not disregard and perpetuate the “insecurity of the people facing persecution in the country of origin or discrimination in their new country or even dying in transit” ( Estevens, 2018, p.19). Such migration policy demands an ‘integrated’ approach, simultaneously tackling the violations of human rights and freedoms, the resolution of conflicts, monetary and organisational development, and environmental protection in a systematic and simultaneous manner ( UNHCR, 1995). It also needs to be set within the wider battle against the feminization of poverty, and the struggle for the full appreciation of diversity and women’ equality and liberation everywhere. Moreover, it has to be embedded within wider struggles for social, economic and environmental justice, on both the micro personal level and the structural global north-south divide. Conversely, the EU’s management of migration through its sustained policy of the externalisation of its borders will only lead to additional ‘Bad Deals’ and ‘Tragic Pacts’ for ‘Women on the Move’.

## Data Availability

No Data associated with this article.

## References

[ref-1] Ager A Strang A : Understanding integration: a conceptual framework. *J Refug Stud.* 2008;21(2):166–191. 10.1093/jrs/fen016

[ref-2] Albrecht C Pérez MH Stitteneder T : The integration challenges of female refugees and migrants: where do we stand? *CESifo Forum.* 2021;22(2):39–46. Reference Source

[ref-3] Ali JS McDermott S Gravel RG : Recent research on immigrant health from statistics Canada’s population surveys. *Can J Public Health.* 2004;95(3):I9–113. 10.1007/BF03403659 15191126 PMC6975564

[ref-4] Akua-Sakyiwah B : Education as cultural capital and its effect on the transitional issues faced by migrant women in the diaspora. *Int Migration & Integration.* 2016;17:1125–1142. 10.1007/s12134-015-0455-8

[ref-5] Amnesty International: No one will look for you.2021. Reference Source

[ref-7] Casajuana E Pintus GJ : Beyond borders, beyond boundaries: a critical analysis of eu financial support for borders control in Tunisia and Libya. The Greens/EFA in the European Parliament,2023. Reference Source

[ref-8] Cheung SY Phillimore J : Gender and refugee integration: a quantitative analysis of integration and social policy outcomes. *J Soc Policy.* 2017;46(2):211–230. 10.1017/S0047279416000775

[ref-9] Council of the European Union: Concept on strengthening EU mediation and dialogue capacities. Brussels,2009. Reference Source

[ref-10] Dağdelen S : A left-wing critique of the migration pact. International Politics and Society, December 7,2018. Reference Source

[ref-11] De Petris A : The new european pact on migration and asylum: pragmatism at all costs threatens long-term credibility. Centro Politiche Europee, December 20,2023. Reference Source

[ref-12] Deacon Z Sullivan C : Responding to the complex and gendered needs of refugee women. *Affilia.* 2009;24(3):272–84. 10.1177/0886109909337401

[ref-13] De Maio J Silbert M Stathopoulos M : Empowering migrant and refugee women: supporting and empowering women beyond five-year post-settlement. (Research Report No. 38). Melbourne: Australian Institute of Family Studies,2017. Reference Source

[ref-14] De Vries W : Oil, arms and emissions: the role of the military in a changing climate.In: A. Dunlap. & A. Brock. (eds): *Enforcing Ecocide.*Palgrave Macmillan, Cham,2022. 10.1007/978-3-030-99646-8_7

[ref-15] Estevens J : Migration crisis in the EU: developing a framework for analysis of national security and defence strategies. *Comp Migr Stud.* 2018;6(1): 28. 10.1186/s40878-018-0093-3 30363805 PMC6182341

[ref-46] European Commission : A new pact on migration and asylum: state of play. Publications Office of the European Union,2023. Reference Source

[ref-16] Fathalla MF Sinding SW Rosenfield A : Sexual and reproductive health for all: a call for action. *Lancet.* 2006;368(9552):2095–2100. 10.1016/S0140-6736(06)69483-X 17161731

[ref-17] Gesemann F : Indicators of integration in education.2006.

[ref-18] Greens/EFA: Fighting for a humane EU migration pact. The Greens/EFA in the European Parliament. (n.d.). Reference Source

[ref-19] Hathaway JC Neve RA : Making international refugee law relevant again: a proposal for collectivized and solution-oriented protection. *Harv Hum Rts J.* 1997;10:115–211. Reference Source

[ref-20] Jaji R : Refugee women and the experiences of local integration in Nairobi, Kenya. Doctoral degree awarded by BIGSAS at Bayreuth University,2009. Reference Source

[ref-21] Keygnaert I Guieu A : What the eye does not see: a critical interpretive synthesis of European Union policies addressing sexual violence in vulnerable migrants. *Reprod Health Matters.* 2015;23(46):45–55. 10.1016/j.rhm.2015.11.002 26718996

[ref-22] Lamberts P : Deal on migration unworkable & solidifies practices that undermine human rights. The Greens/EFA in the European Parliament, December 20,2023. Reference Source

[ref-23] Lawrence J Kearns R : Exploring the ‘fit’ between people and providers: refugee health needs and health care services in Mt Roskill, Auckland, New Zealand. *Health Soc Care Community.* 2005;13(5):451–461. 10.1111/j.1365-2524.2005.00572.x 16048533

[ref-24] Lochan A Fortier Z Raby B : Global compact for migration: gender responsiveness, implementation and Canada’s role. In: Balsillie School of International Affairs. *Strengthening the Rules-Based International Order*,2019;35–39. Reference Source

[ref-25] Lokshin M Olters JP : Build cities, not camps: a proposal for addressing refugee crisis. Brookings, September 4,2022. Reference Source

[ref-26] Miller KE Rasmussen A : War exposure, daily stressors, and mental health in conflict and post-conflict settings: bridging the divide between trauma-focused and psychosocial frameworks. *Soc Sci Med.* 2010;70(1):7–16. 10.1016/j.socscimed.2009.09.029 19854552

[ref-28] Mwenyango H : Gendered dimensions of health in refugee situations: an examination of sexual and gender-based violence faced by refugee women in Nakivale refugee settlement, Uganda. *Int Soc Work.* 2023;66(4):1247–1261. 10.1177/00208728211003973

[ref-50] Namak S Aboud Syriani F Singer M : Barriers to education and employment among Arabic speaking refugee and immigrant women. *Int J Migr Health Soc Care.* 2022;18(2):125–138. 10.1108/IJMHSC-03-2021-0024

[ref-29] Nicolle H : Digital livelihoods for refugees - examples, challenges and recommendations. The Dialogue on Tech and Migration, DoT.Mig,2022. Reference Source

[ref-30] Ohene-Bekoe S : Exploring refugee women’s access to health care services: the first year.A Thesis submitted to the College of Graduate and Postdoctoral Studies in partial fulfilment of the requirements for the Degree of Master of Science in the Department of Community Health and Epidemiology, University of Saskatchewan, Saskatoon,2017. Reference Source

[ref-31] Pertek S Phillimore J Alidu L : Sexual and gender-based violence and refugees: the impacts of and on integration domains. *Iris Working Paper Series, No. 28/2018*. Institute for Research into Superdiversity University of Birmingham,2018. Reference Source

[ref-32] Peters ME : Open trade, closed borders immigration in the era of globalization. *World Politics.* 2015;67(1):114–154. 10.1017/S0043887114000331

[ref-33] Ramazani U : Building meaningful refugee participation into protection policymaking. Migration Policy Institute,2023. Reference Source

[ref-34] Russell AM : “Victims of trafficking”: the feminisation of poverty and migration in the gendered narratives of human trafficking. *Societies.* 2014;4(4):532–548. 10.3390/soc4040532

[ref-35] Setia MS Quesnel-Vallee A Abrahamowicz M : Access to health care in Canadian immigrants: a longitudinal study of the National Population Health Survey. *Health Soc Care Community.* 2011;19(1):70–79. 10.1111/j.1365-2524.2010.00950.x 21054621 PMC3762743

[ref-36] Shaw D : Access to sexual and reproductive health for young people: bridging the disconnect between rights and reality. *Int J Gynaecol Obstet.* 2009;106(2):132–136. 10.1016/j.ijgo.2009.03.025 19535075

[ref-37] Snel E Bilgili Ö, Staring R : Migration trajectories and transnational support within and beyond Europe. *J Ethnic Migr Stud.* 2021;47(14):3209–3225. 10.1080/1369183X.2020.1804189

[ref-47] Standke-Erdmann M : Intersectionality and Refugee Women: The Shortcomings of the EU Pact on Migration and Asylum from an Intersectional Perspective.2021. Reference Source

[ref-38] Tavara L : Sexual violence. *Best Pract Res Clin Obstet Gynaeco.* 2006;20(3):395–408. 10.1016/j.bpobgyn.2006.01.011 16564226

[ref-39] UN: Global compact for safe, orderly and regular migration. UN Doc. A/RES/73/195, December 19,2018. Reference Source

[ref-48] UN General Assembly: Universal Declaration of Human Rights. United Nations, 217 (III) A, Paris,1948. Reference Source

[ref-41] UNHCR: Action against sexual and gender-based violence: an updated strategy.2011. Reference Source

[ref-42] UNHCR: The state of the world’s refugees: in search of solutions.Oxford University Press,1995. Reference Source

[ref-40] UNICEF: Empowering syrian refugee women in jordan: a proposal prepared for UNICEF next generation.2017. Reference Source

[ref-43] Vu A Adam A Wirtz A : The prevalence of sexual violence among female refugees in complex humanitarian emergencies: a systematic review and meta-analysis. *PLoS Curr.* 2014;6. 24818066 10.1371/currents.dis.835f10778fd80ae031aac12d3b533ca7 PMC4012695

[ref-44] Wessendorf S Phillimore J : New migrants’ social integration, embedding and emplacement in superdiverse contexts. *Sociology.* 2018;53(1). 10.1177/0038038518771843

[ref-45] Women for Women International: The number of forcibly displaced people has reached a staggering number—over 100 million according to reliefweb—and the number only continues to rise. June 5,2023. Reference Source

