# Supplementary material for: Bad Deals and Tragic Pacts: The impact of the EU’s management of migration through the externalisation of its borders on ‘Women on the Move’
Source: Open Res Eur. 2024 Aug 28;4:187. [Version 1] doi: 10.12688/openreseurope.18388.1 (PMC11550392; doi:10.12688/openreseurope.18388.1)
Supplement: Peer review history

# Reviewer report: Reviewer response for version 1

**DOI:** 10.21956/openreseurope.19868.r44280

**Published:** 2024-11-09

**Authors:** Valeria Bello

**Affiliations:** 1 Ramón Llull University, Barcelona, Spain

The topic that the article initially proposes is very relevant and would be a very good contribution to the literature on migration and women's insecurities. However, the work would need revision in the way it is developed. This is mostly due to the fact that what the article promises in the abstract and in the introduction is not discussed in-depth in the following section. The article very relevantly starts with considering that the EU-Turkey Deal paved the way for further agreements with third countries and this  exposes women migrants to further vulnerabilities due to restrictive policies in those countries, adding additional risks of abuses and gender-based violence. However, after the introduction, which is indeed very well written and interesting, the following section titled "female integration and wellbeing" does not discuss those added vulnerabilities in third countries to which the EU externalized migration management, but rather the section describes more generally the challenges that women could experience in every destination country, even in the EU. In itself, this section is also well written and well discussed but it does not deal with the topic suggested in the very beginning. Here, the author should discuss what are the added vulnerabilities that women face in these third countries where the EU has extended the externalization of migration, such as Libya and Tunisia, as said in the abstract.

 The section on intersectionality and the following one on embodied enquiry are crucial for the analysis and I would suggest referring to these issues more transversally in the article.

 Also, in the abstract and in the last section, it is written that the EU Pact on Migration and Asylum was adopted in 2023, but it has indeed been adopted by the EU Parliament and the European Council in 2024. In December 2022 and in December 2023 there were provisional agreements between the EU Member States on different regulations and directives that form the EU Pact on Migration and Asylum proposed by the EU Commission in 2022. Also, the pact's title is not "New Pact on Migration and Asylum" but it is "EU Pact on Migration and Asylum". So, it would be fine to say the new "EU Pact on Migration and Asylum" but not the "New Pact on Migration and Asylum", because it is not its title and secondly, there has never been a previous one signed. So, more attention to such details is indispensable if the "EU Pact on Migration and Asylum" is to be included in the analysis.

 The relative section on the EU Pact also presents several paragraphs that start with the same sentence "The Pact has been severely criticised". I would advise to formulate the sentences differently, using synonyms, in order to have a more elegant linguistic style.

 Another element to be highlighted is the uneven distribution of the analysis: the last section on the "EU Pact on Migration and Asylum" is much longer than the previous ones, unevenly skewing the analysis on the Pact itself and moving the attention away from the topic initially proposed.

 In addition to this, the argument that the EU-Turkey deal has paved the way for additional externalization of migration is not proved in any of the previous sections with a concrete analysis. It therefore remains an argument that the article fails to develop in the analysis.

 The section on the EU Pact on Migration and Asylum also reads as a literature review and not as a contribution to the literature. It is not adding any specific original analysis that would contribute to the current literature. 

 I would suggest the following changes:

 1) To revise the section on female integration and wellbeing and include an in-depth discussion of what are the added vulnerabilities entailed for women by those pacts mentioned in the abstract.

 2) To prove the argument that the EU-Turkey deal paved the way for further agreements.

 3) To include more connection throughout the paper and particularly in the analysis of the specific case studies on the issue of intersectionality and embodied inquiry.

 4) To revise the section on the EU Pact so as to avoid that it reads only like a review of current existing literature on it and add a specific examination of some parts of the Pacts in line with what is suggested previously in the article, such as the issues of heterogeneity, intersectionality and the embodied inquiry.

Is the argument persuasive and supported by appropriate evidence?

No

Is the work clearly and cogently presented?

No

Is the topic of the essay discussed accurately in the context of the current literature?

Yes

Does the essay contribute to the cultural, historical, social understanding of the field?

Partly

Reviewer Expertise:

Migration and Security; Intersectionality; Inclusion; Prejudice; Racism.

I confirm that I have read this submission and believe that I have an appropriate level of expertise to confirm that it is of an acceptable scientific standard, however I have significant reservations, as outlined above.

# Reviewer report: Reviewer response for version 1

**DOI:** 10.21956/openreseurope.19868.r44279

**Published:** 2024-10-31

**Authors:** Christian Kaunert

**Affiliations:** 1 University of South Wales, Pontypridd, Wales, UK

Bad Deals and Tragic Pacts: The impact of the EU’s management of migration through the externalization of its borders on ‘Women on the Move

**Major Revisions**

 This could become a fascinating piece given that the author demonstrates empirical and theoretical knowledge on the topic of EU migration management, which could eventually lead to a future publication. However, I believe there are some shortcomings that, in my view, would prevent outright approval. Therefore, I recommend requesting major revisions considering my comments below.

 Firstly, I appreciate the topic of the article. Indeed, this subject is under-researched and should be further explored in future articles. The author has uncovered something exciting and indeed under-researched.

 There are some shortcomings that need addressing:

  

- the article needs rebalancing: at present, too much space is dedicated to minute points of the empirical dataset at various points in the article, to the detriment of bringing in conceptual analysis. In this process, the author's own aims become hidden. The article should maintain a tighter focus on its primary aim.
- The introduction is long-winded at the beginning and should state the main aim more swiftly. Why is this topic interesting, despite some criticisms one could raise? What contributions does this article make? Subsequently, the author should clarify the benefits of his/her approach.
- The methodology could be explained more easily to non-specialists regarding that approach. What methodology has the author employed to support the article's claims? More methodological clarity is needed.
- The author must engage more with the EU asylum and migration literature, which would significantly strengthen the article.
- Specifically, there is an issue of under-referencing: much has been written about EU asylum and immigration policy, and the article does not articulate its contribution through the existing literature on this topic. Concretely, the following authors need to be included:
  - El-Enany, N. (2008) [Ref 1]
  - Geddes, A. (2008) [Ref 2] 
  - Guiraudon, V. (2003) [Ref 3]
  - Kaunert, C. (2009) [Ref 4] 
  - Kaunert, C. (2010) [Ref 5]
  - Kaunert C. & S. Léonard (2012): [Ref 6]
  - [Ref 7]
  - Léonard S. & C. Kaunert (2019): [Ref 8]
  - Léonard S. & C. Kaunert (2020): [Ref 9]
  - Levy, C. (2010) [Ref 10] 

In addition, there is also a minor point to be revised: the article's overall argument could be signposted more clearly to ensure the reader understands the significance of the points raised in the article.

 Nonetheless, despite some criticisms, the article could and should be revised along the points mentioned above. I encourage the author to work on this. Congratulations on finding such an interesting topic! I very much enjoyed reading the article!

Is the argument persuasive and supported by appropriate evidence?

Partly

Is the work clearly and cogently presented?

Yes

Is the topic of the essay discussed accurately in the context of the current literature?

Partly

Does the essay contribute to the cultural, historical, social understanding of the field?

Yes

Reviewer Expertise:

EU asylum and migration

I confirm that I have read this submission and believe that I have an appropriate level of expertise to confirm that it is of an acceptable scientific standard, however I have significant reservations, as outlined above.

## References

[1] : Who is the New European Refugee. *https://papers.ssrn.com/sol3/papers.cfm?abstract_id* .2007;

[2] : Immigration and European Integration: Towards Fortress Europe. *https://manchesteruniversitypress.co.uk/9780719074660/* .2008;

[3] : The constitution of a European immigration policy domain: a political sociology approach. *Journal of European Public Policy* .2003;10(2) : 10.1080/1350176032000059035 263-282 10.1080/1350176032000059035

[4] : Liberty versus Security? EU Asylum Policy and the European Commission. *Journal of Contemporary European Research* .2009;5(2) : 10.30950/jcer.v5i2.172 148-170 10.30950/jcer.v5i2.172

[5] : European Internal Security: Towards Supranational Governance in the Area of Freedom, Security and Justice. *https://discovery.dundee.ac.uk/en/publications/european-internal-security-towards-supranational-governance-in-th* .2010;

[6] : The development of the EU asylum policy: venue-shopping in perspective. *https://www.academia.edu/61767484/The_development_of_the_EU_asylum_policy_venue_shopping_in_perspective* .2012;

[7] : The European Union Asylum Policy after the Treaty of Lisbon and the Stockholm Programme: Towards Supranational Governance in a Common Area of Protection?. *Refugee Survey Quarterly* .2012;31(4) : 10.1093/rsq/hds018 1-20 10.1093/rsq/hds018

[8] : The development of the EU asylum policy: venue-shopping in perspective. *https://www.routledge.com/Refugees-Security-and-the-European-Union/Leonard-Kaunert/p/book/9780367727239?srsltid* .2020;

[9] : The securitisation of migration in the European Union: Frontex and its evolving security practices. *Journal of Ethnic and Migration Studies* .2022;48(6) : 10.1080/1369183X.2020.1851469 1417-1429 10.1080/1369183X.2020.1851469

[10] : Refugees, Europe, Camps/State of Exception: "Into The Zone", the European Union and Extraterritorial Processing of Migrants, Refugees, and Asylum-seekers (Theories and Practice). *Refugee Survey Quarterly* .2010;29(1) : 10.1093/rsq/hdq013 92-119 10.1093/rsq/hdq013

# Reviewer report: Reviewer response for version 1

**DOI:** 10.21956/openreseurope.19868.r43803

**Published:** 2024-10-15

**Authors:** Stefania Panebianco

**Affiliations:** 1 University of Catania, Catania, Italy

This paper addresses a relevant issue of EU migration politics and raises interesting questions related to a specific category of irregular migrants: 'women on the move'. The article focuses upon specific risks girls and women are faced with when embarking in such dangerous journeys and suggests the establishment of the right to asylum for refugee women and girls. This reviewer acknowledged the high potential of this article. 

 The article is clearly policy-oriented and offers a critical review of the existing norms and regulations of a complex policy that - as it stands - does not guarantee women on the move and denies humanitarian treatments. It starts with a criticism of existing international refugee law and its ineffective humanitarian protection of those in need (women in particular) and then critically explores the external dimension of the European migration and asylum policy.

 The use of data to strengthen the author’s argument is convincing.

 What remains less convincing though, is the selection of the EU-Turkey deal as a key example of inhuman and degrading treatment, without providing specific empirical analysis. Moreover, there is a temporal gap in the analyzed documents, passing from the EU-Turkey deal to the New Pact on Migration and Asylum. What is less clear in the document selection is the relevance of the gender-issue in the EU externalization of migration management. In the second part of the paper, in fact, considerations on the externalization of EU borders and, more generally, human dignity and humanitarian concerns put in the shadow the specific focus on ‘women on the move’.

 Nevertheless, the argument is strong and suggests clear paths for further analysis and more effective dimensions of international protection.

Is the argument persuasive and supported by appropriate evidence?

Yes

Is the work clearly and cogently presented?

Yes

Is the topic of the essay discussed accurately in the context of the current literature?

Yes

Does the essay contribute to the cultural, historical, social understanding of the field?

Yes

Reviewer Expertise:

Mediterranean migration; external dimension of the European migration and asylum policy; EU externalization of migration management

I confirm that I have read this submission and believe that I have an appropriate level of expertise to confirm that it is of an acceptable scientific standard.
